# Learning analytics and the future of postgraduate medical training

**DOI:** 10.1007/s11845-024-03702-9

**Published:** 2024-05-24

**Authors:** George Shorten

**Affiliations:** 1 The College of Anaesthesiologists of Ireland, Dublin, Ireland; 2 University College Cork, Cork, Ireland; 3 Insight SFI Research Centre for Data Analytics, Dublin, Ireland

**Keywords:** Learning analytics · Medical training · Postgraduate

## Abstract

Confronted by the many barriers and deficiencies which currently face those responsible for the training of doctors, the concept of a logic model applied in real time may seem aspirational. However, several of the necessary of logic-based practices are already in place — these include quantified training effect and performance, learning analytics, and applied reflective practice. A nationally or internationally co-ordinated effort is required to harness these disciplines (which currently exist disparately) to create a sustainable and effective training system which is adaptive to its own performance and to society’s changing needs. This will mean making better use of the data currently being generated by and around training, and its presentation in a timely and comprehensible form to the person(s) who is responsible, prepared, and able to use it to best effect.

“From the earliest times, medicine has been a curious blend of superstition, empiricism, and that kind of sagacious observation which is the stuff out of which ultimately science is made.” Flexner 1910 [1]

From ancient times, the “training” of medical practitioners has been diverse and subject to few or no formal standards until the nineteenth century. In the early 1890s, William Osler introduced a structured “residency” programme for the training of doctors at Johns Hopkins Hospital [2, 3]. This time-based, apprenticeship model formed the basis of much postgraduate medical training in North America, Europe, and elsewhere during the twentieth century [2]. At the turn of the twenty-first century, the US Accreditation Council for Graduate Medical Education and other regulators introduced the move to competency-based medical education [4]. The rationale was clear: if the medical practitioners were to practice safely and well, the role of training was to ensure that they were able to do so. The duration spent in training was no longer the primary or sole measure required to graduate to independent practice. There is no doubt that the move from time based to competency-based training was influenced by the direction offered by national medical organisations and the realisation that medical errors and preventable harm (associated with healthcare) occurred commonly [5].

In 2010, the Lancet Commission (on Education of Health Professionals for the 21st Century) stated unambiguously that all was not well — “a slow-burning crisis is emerging in the mismatch of professional competencies to patient and population priorities because of fragmentary, outdated, and static curricula producing ill-equipped graduates from underfinanced institutions” [6]. The Commission recommended a systems-based approach to the reform of training which would require buy in from the many stakeholders (educators, students and young healthcare workers, professional bodies, universities, non-governmental organisations, international agencies, donors, and foundations) [6]. It also identified the sharing of learning using metrics as a means of evaluation and research as a key enabler [6].

Our recent Consensus Statement on the meaning, value, and utility of Training Programme outcomes strongly endorsed the need for a systems-based approach to postgraduate medical training [7]. Of course, the complexity and interactions of the systems in question (policy, funding, regulation, teaching, training, and learning) present a major challenge to implementing such an approach successfully. To address this challenge, we propose that a logic model such as that described by Van et al. [8] be used. This may not be as “aspirational” as it appears at first sight; several components of a logic model already operate in different countries. If, in any jurisdiction, genuine stakeholder “buy in” can be achieved and a collaborative governance structure put in place; then, the keys to operating such a system are the access to and use of data.

To operate a logic model for the postgraduate training of doctors, it would be necessary to (i) collect *a*ccurate and relevant data and (ii) to present them in a timely and comprehensible form (iii) to a person or entity who/which is prepared and able to respond to them.

To examine this possibility from the point of view of a medical trainee, one might ask:

What types of data are relevant to me and my training ?

What does *comprehensible* mean for me ?

And am I either prepared and able to respond in a way that will benefit my training?

Taking each of these in turn, the quality of one’s professional performance is relevant and critically important to any medical trainee. It is strange that, at present, no internationally accepted norm exists for generating this information. Ideally, this would require accurate and reliable quantification of performance of clinical skills and other professional tasks. The definition and capture of specific observable behaviours or “metrics” provide a means of supporting deliberate practice and quantitative assessment of performance with reference to a standard. Such “metrics-based” training and assessment are quite widely employed in surgical training programmes [9]. Recently, one form of metrics-based training, namely, proficiency-based progression training has been shown to decrease the number errors made by trainees (by 60% on average), and to consistently transfer training benefit from the simulation to the clinical environment [10]. For the operation of a logic model, such training provides a data stream of valid, reliable, and *accurate information which is critically relevant to an individual trainee* but also in aggregate form to a training programme director.

Learning analytics refers to a relatively new discipline and has been defined as “the measurement, collection, analysis, and reporting of data about learners and their contexts, for the purposes of understanding and optimizing learning and the environments in which it occurs” [11]. Broadly, the analysis performed can enable (i) summary, including visualisations, of the relevant data; (ii) some form of interpretation or meaning extraction; and (iii) prediction of future events such as achievement of learning outcomes. Increasingly learners can access summary of their learning and engagement activities using a dashboard and receive advice through a personalised recommender system. Thus, LA offers the potential to present useful information to medical trainees which is *comprehensible*, and relevant to them as individual, adult, professional learners. It can also signpost suitable resources which are specific to a particular trainer at a particular point in their development. Learning analytics will certainly be employed in the future to provide suitable information in comprehensible form to the right person (i.e., healthcare professional or trainee) at the right time.

The extent to which one responds positively to information about one’s professional performance may be determined by one’s reflective ability. In medicine, convincing evidence indicates that reflective practice results in improvements in self-directed learning, improved motivation, and enhanced quality of patient care (e.g., accuracy of diagnosis) [12, 13]. Using a framework for reflection in medical practice, a well-designed dashboard could support “Reflection on Action” in particular [14]. In this way, a medical trainee who is *prepared and able* to reflect considers actions and decisions already taken in order to determine what worked well and what could be improved upon. Data use optimisation could provide information about the actions and decisions taken by others as well as oneself (for instance from published literature, aggregate training, or registry datasets).

The employment of learning analytics for training and education will present important challenges to training bodies, regulators, and to trainers and to trainees. These have been well described by Ten Cate et al. [15] and relate to such issues as learner privacy and data protection, lesser trainee autonomy in how learning is approached, and the potential to create additional workload for trainers with consequent lessening of enthusiasm for teaching. The explicit enunciation of these risks is valuable in itself; they need to be managed proactively as the implementation of learning analytics for health professional training occurs. For instance, Drachsler and Greller’s DELICATE checklist offers one feasible approach to ensuring that learners’ privacy is protected while learning advantages are retained [16]. One could foresee a system which, through analysis of aggregate data, identifies a set of learning practices which are associated with optimal outcome (perhaps in progression reviews or examination results). If presented inappropriately, this information could incentivize learners to adopt these practices in an unthinking way and could drive unhealthy “a one size fits all” behaviours. Thus, the actionable outputs of learning analytics will require careful consideration of how, when and by whom they are provided to learners. The discipline specific expertise of trainers and supervisors will be one important element of how learning analytics outputs are interpreted for and with trainees.

A clear need exists within medical training to directly address the tensions that exist between healthcare service delivery and the education of future doctors. Given the extent of health and healthcare interconnection with every aspect of society, a systems-based approach to medical training is necessary. In the context of Irish healthcare, key stakeholders would include the Health Service Executive, the Medicla Council of Ireland, and other regulatory bodies, the accredited training bodies and patient advocacy organisations. To operate such an approach, one feasible option is to develop a logic model within which many stakeholders (including patients and their families) provide access, analyse and share data efficiently. Confronted by the many barriers and deficiencies which currently face those responsible for the training of doctors, the concept of a logic model applied in real time may seem aspirational. However, several of the necessary of logic-based practice are already in place — these include quantified training effect and performance, learning analytics, and applied reflective practice. A nationally or internationally co-ordinated effort is required to harness these disciplines (which currently exist disparately) to create a sustainable and effective training system which is adaptive to its own performance and to society’s changing needs. This will mean making better use of the data currently being generated by and around training, and its presentation in a timely and comprehensible form to the person(s) who is responsible, prepared, and able to use it to best effect.
